# DISPARITIES OF DENTAL CARE AND CONSEQUENCE OF POOR ORAL HEALTH AMONG OLDER ADULTS IN THE U.S.

**DOI:** 10.1093/geroni/igz038.2257

**Published:** 2019-11-08

**Authors:** Yanyan Wu, Wei Zhang, Bei Wu

**Affiliations:** 1 University of Hawai’i at Mānoa, Honolulu, Hawaii, United States; 3 New York University, New York, New York, United States

## Abstract

Oral health is an essential part of staying healthy. Neglect of dental care may lead to tooth decay/ loss, poor nutrition, and affects individuals’ quality of life. Over the past decades, dental care utilization has risen considerably, however, racial/ethnic and socioeconomic disparities still persist in the U.S. Additionally, poor oral health is a contributing factor to, and a consequence of chronic diseases such as cognitive impairment, diabetes and cardiovascular disease. Faced with the complex and intertwined health and social challenges, it’s imperative to understand the disparities of dental care utilization and the relationships among oral health and chronic diseases so that effective policies and preventions can be implemented to improve quality of care. In this symposium, we present findings for older adults from diverse racial/ethnic populations in the U.S. We begin with results from two national-wide trend analyses: a 15-year review of dental care utilization and the evaluation of dental care performance over a 16-year period in nursing homes. The next study presents the barriers of dental care utilization in Hawaii. Finally, we present results of the negative effects of diabetes and poor oral health on cognitive function. Our studies address the disparities of dental care utilization among minority and under-represented ethnic groups as well as the connections between oral health and chronic conditions. Our results are helpful in educating policy makers and health practitioners about how to improve dental care and how dental care can be effectively integrated into chronic disease prevention and health promotion activities.

